# Dietary Iron Overload and *Hfe^−/−^* Related Hemochromatosis Alter Hepatic Mitochondrial Function

**DOI:** 10.3390/antiox10111818

**Published:** 2021-11-16

**Authors:** Christine Fischer, Chiara Volani, Timea Komlódi, Markus Seifert, Egon Demetz, Lara Valente de Souza, Kristina Auer, Verena Petzer, Laura von Raffay, Patrizia Moser, Erich Gnaiger, Guenter Weiss

**Affiliations:** 1 Department of Internal Medicine II, Medical University of Innsbruck, Anichstrasse 35, 6020 Innsbruck, Austria; christine.fischer@i-med.ac.at (C.F.); chiara.volani@eurac.edu (C.V.); markus.seifert@i-med.ac.at (M.S.); egon.demetz@i-med.ac.at (E.D.); lara.valente@i-med.ac.at (L.V.d.S.); kristina.auer@labordiagnostik.tirol (K.A.); verena.petzer@i-med.ac.at (V.P.); laura.von-raffay@i-med.ac.at (L.v.R.); 2 Oroboros Instruments, Schöpfstrasse 18, 6020 Innsbruck, Austria; timea.komlodi@oroboros.at (T.K.); erich.gnaiger@oroboros.at (E.G.); 3 Christian Doppler Laboratory for Iron Metabolism and Anemia Research, Medical University of Innsbruck, Anichstrasse 35, 6020 Innsbruck, Austria; 4 Department of Pathology, Innsbruck University Hospital, Anichstrasse 35, 6020 Innsbruck, Austria; patrizia.moser@tirol-kliniken.at

**Keywords:** hemochromatosis, iron overload, reactive oxygen species, mitochondria, mitochondrial respiration, liver, fatigue

## Abstract

Iron is an essential co-factor for many cellular metabolic processes, and mitochondria are main sites of utilization. Iron accumulation promotes production of reactive oxygen species (ROS) via the catalytic activity of iron species. Herein, we investigated the consequences of dietary and genetic iron overload on mitochondrial function. C57BL/6N wildtype and *Hfe^−/−^* mice, the latter a genetic hemochromatosis model, received either normal diet (ND) or high iron diet (HI) for two weeks. Liver mitochondrial respiration was measured using high-resolution respirometry along with analysis of expression of specific proteins and ROS production. HI promoted tissue iron accumulation and slightly affected mitochondrial function in wildtype mice. Hepatic mitochondrial function was impaired in *Hfe^−/−^* mice on ND and HI. Compared to wildtype mice, *Hfe^−/−^* mice on ND showed increased mitochondrial respiratory capacity. *Hfe^−/−^* mice on HI showed very high liver iron levels, decreased mitochondrial respiratory capacity and increased ROS production associated with reduced mitochondrial aconitase activity. Although *Hfe^−/−^* resulted in increased mitochondrial iron loading, the concentration of metabolically reactive cytoplasmic iron and mitochondrial density remained unchanged. Our data show multiple effects of dietary and genetic iron loading on mitochondrial function and linked metabolic pathways, providing an explanation for fatigue in iron-overloaded hemochromatosis patients, and suggests iron reduction therapy for improvement of mitochondrial function.

## 1. Introduction

Iron is an essential trace element for life, as it represents a crucial cofactor for key metabolic enzymes involved in vital processes such as DNA replication, hormone synthesis and mitochondrial bioenergetics [1,2,3]. In mitochondria, iron is required for heme synthesis, iron sulfur (Fe-S) cluster synthesis and oxidative phosphorylation [4,5,6]. Complexes I, II and III of the electron transfer system (ETS) contain Fe-S clusters essential for electron transfer and subsequent production of adenosine triphosphate (ATP) [7,8,9]. Substrates, mainly nicotinamide adenine dinucleotide (NADH), originate from the tricarboxylic acid (TCA) cycle depending on the activities of enzymes such as mitochondrial aconitase (m-aconitase, ACO2), which converts citrate to isocitrate. m-Aconitase expression increases with iron availability [10,11]. In vitro data have shown that iron availability affects mitochondrial function and cellular oxygen consumption by modulating the expression of TCA cycle enzymes, including translational control of m-aconitase expression [2,12]. In contrast, cytosolic aconitase (c-aconitase, ACO1) contains an Fe-S cluster and acts depending on intracellular iron levels as an iron regulatory protein (IRP-1) involved in the posttranscriptional control of mRNA expressions coding for cellular iron transport (transferrin receptor 1 (TfR1), divalent metal transporter 1 and ferroportin (FPN)), iron storage (ferritin) or iron metabolization genes (m-aconitase, amino-levulinate synthase) [1,13]. Iron metabolism needs to be tightly regulated to avoid iron deficiency with negative effects on metabolic functions and to prevent cellular iron accumulation, as this metal can catalyze the formation of reactive oxygen species (ROS) by the Fenton/Haber–Weiss reaction [2,6,11,14].

Iron overload can emerge on the basis of genetic defects in several regulatory iron genes, termed as primary iron overload or hereditary hemochromatosis [15,16]. In addition, iron accumulation can develop as a consequence of dyserythropoiesis resulting from inborn errors of hemoglobin synthesis such as thalassemia or repeated blood transfusions, both termed as secondary iron overload [11]. As no efficient iron excretion mechanism exists, iron accumulates in parenchymal organs such as liver, heart and endocrine tissues, where it promotes toxic radical formation, thus resulting in cellular damage and organ dysfunction over time [14,17,18,19]. The most common primary iron overload disease is hereditary hemochromatosis type I (HH), characterized by a missense mutation in the non-classical major histocompatibility complex (MHC) class I protein HFE (C282Y) that forms a complex with TfR1 [16,20,21,22]. This mutation causes reduced plasma levels of the hormone hepcidin, which regulates iron homeostasis, thus resulting in increased duodenal iron absorption and parenchymal iron overload over time [16,21,23].

Limited information is available on mitochondrial function in genetic hemochromatosis and secondary iron overload. For the latter model, dietary iron loading of mice is an established model leading to tissue iron accumulation over time, comparable to the situations seen in humans with secondary iron loading [24,25]. Previous investigations have demonstrated mitochondrial dysfunction and decreased mitochondrial oxygen consumption due to increased ROS formation and peroxidative injuries as well as decreased activity of manganese superoxide dismutase (MnSOD) in isolated mitochondria but not in whole tissue ex vivo [26,27,28]. In the present study, we investigated whether dietary and/or genetic iron overload affects mitochondrial function and alters metabolic pathways in the liver.

## 2. Materials and Methods

### 2.1. Animals

Wildtype C57BL/6N mice (Wt) and C57BL/6N mice with a knockout of the *Hfe* gene (*Hfe^−/−^*) [29] were bred in the central animal facility at the Medical University of Innsbruck (Innsbruck, Austria) under specific pathogen-free conditions. All animal experiments were performed in accordance with national and European guidelines and were reviewed and authorized by the committee on animal experiments (Federal Ministry of Science and Research, BMWFW-66.011/0074-WF/II/3b/2014 and 2020-0.448.830). At the age of 42 weeks, mice were randomly assigned either to the group receiving a standard chow diet (ND; 180 mg iron/kg, Sniff, Soest, Germany) or a standard chow diet supplemented with 25 g/kg of carbonyl iron (HI, Sniff, Soest, Germany) ad libitum for two weeks. This or similar feeding strategies have previously been applied to induce short-term systemic iron overload in mice [14,30,31]. Both diets contained all compounds necessary for mice [32]. After two weeks, the animals were sacrificed; plasma and organs were collected for further analyses.

### 2.2. High-Resolution Respirometry

All measurements were performed as described [7,14,33]. Briefly, mitochondrial respiration was performed using the Oxygraph-2k (O2k, Oroboros Instruments, Innsbruck, Austria). Fresh mouse liver tissue samples were collected and homogenized. All measurements were performed under normoxic conditions in the mitochondrial respiration medium MiR05-Kit (Oroboros Instruments, Innsbruck, Austria) containing 0.5 mM ethylene glycol tetraacetic acid (EGTA), 3 mM magnesium chloride (MgCl_2_), 60 mM lactobionic acid, 20 mM taurine, 10 mM monopotassium phosphate (KH_2_PO_4_), 20 mM 4-(2-hydroxyethyl)-1-piperazineethanesulfonic acid (HEPES), 110 mM D-sucrose, 1 g/l essentially fatty acid-free bovine serum albumin (BSA, Sigma-Aldrich, St. Louis, MO, USA). Manual titrations of substrates, uncoupler and inhibitors were performed using Hamilton syringes (customized for Oroboros Instruments, Hamilton Central Europe, Giarmata, Romania). The following substrate-uncoupler-inhibitor titration (SUIT) protocol was used (Supplemental Figure S1): non-phosphorylating LEAK respiration was assessed by injecting pyruvate (P, 5 mM) and malate (M, 2 mM) as NADH (N)-linked substrates in the absence of adenylates, PM*_L_*; OXPHOS capacity was measured by adding adenosine diphosphate (ADP, 2.5 mM) at kinetically saturating concentration, PM*_P_*; cytochrome c (c, 10 µM) was added to test for the mitochondrial outer membrane integrity, PM*_cP_*; stepwise titrations of the protonophore carbonyl cyanide m-chloro phenyl hydrazine (U, 0.5 µM steps) allowed to reach the maximal electron transfer (ET) capacity, PM*_E_*; glutamate (10 mM) was added as an N-linked substrate in non-coupled ET state, PGM*_E_*; by addition of succinate (S, 10 mM) the simultaneous action of N-linked substrates and succinate with convergent electron flow in the NS-pathway for reconstitution of the TCA cycle function was measured, PGMS*_E_*; titration of octanoylcarnitine (Oct, 0.5 mM) enabled the simultaneous action of F- and N-linked substrates and S with convergent electron flow in the FNS pathway for reconstitution of TCA cycle function and additive or inhibitory effect of F-linked substrate Oct to support fatty acid oxidation, OctPGMS*_E_*; Complex I inhibition by rotenone (Rot, 0.5 µM) induced succinate-linked ET capacity, S*_E_*; titration of glycerophosphate (Gp, 10 mM) provided simultaneous action of convergent S- and Gp-linked electron entry in the SGp-pathway, SGp*_E_*; injection of antimycin A (2.5 µM) blocked Complex III and induced the state of residual oxygen consumption, ROX. Data analysis was performed using the software DatLab 7.4 (Oroboros Instruments).

### 2.3. Plasma and Total Tissue Iron

Iron concentration in frozen plasma samples was measured using QuantiChrom Iron Assay Kit (BioAssay Systems, Hayward, CA, USA) according to the manufacturer’s instructions. Tissue iron determination from snap-frozen liver samples was performed as described [14,34]. After acidic hydrolysis at 65 °C for 24 h, the iron content was measured using a colorimetric staining solution containing sodium acetate and bathophenanthroline disulfonic acid. Total iron content was normalized by protein content.

### 2.4. Prussian Blue Staining

Prussian blue staining was performed as described [35].

### 2.5. Isolation of Mitochondria

Crude mitochondria were isolated according to Wieckowski et al. [36]. Briefly, snap-frozen liver tissue was homogenized in isolation buffer 1 (225 mM mannitol, 75 mM sucrose, 0.5% BSA (fatty acid free), 0.5 mM EGTA, 30 mM Tris–HCl pH 7.4) and centrifuged twice at 740× *g* for 5 min at 4 °C. The pellet containing the nucleus fraction and cell debris was discarded. The supernatant containing the cytosolic, the mitochondrial and the mitochondria-associated membrane fractions was centrifuged at 9000× *g* for 10 min at 4 °C and resuspended in isolation buffer 2 (225 mM mannitol, 75 mM sucrose, 0.5% BSA (fatty acid free), 30 mM Tris–HCl pH 7.4). After centrifugation at 10,000× *g* for 10 min at 4 °C, the crude mitochondria pellet was resuspended in isolation buffer 3 (225 mM mannitol, 75 mM sucrose, 30 mM Tris–HCl pH 7.4) and centrifuged again at 10,000× *g* for 10 min at 4 °C. Purity of the cytosolic and mitochondrial fractions was ensured by Western blot (Supplemental Figure S2) and tissue iron measurement was performed as described in Section 2.3.

### 2.6. RNA Extraction and Quantitative Real-Time PCR

Liver total RNA was extracted from snap-frozen liver samples using TRI reagent (Sigma-Aldrich, St. Louis, MO, USA) according to the manufacturer’s protocol. After reverse transcription, mRNA expression was analyzed as described [37]. The following primers were used: *Hepcidin* forward GGCAGACATTGCGATACCAAT, *Hepcidin* reverse TGCAACAGATACCACACTGGGAA, *Hepcidin* probe CCAACTTCCCCATCTGCATCTTCTGC, *Glucuronidase beta* (*Gusβ*) forward CTCATCTGGAATTTCGCCGA, *Gusβ* reverse GGCGAGTGAAGATCCCCTTC, *Gusβ* probe CGAACCAGTCACCGCTGAGAGTAATCG. Quantitative real-time PCR reactions were performed on the CFX96 PCR System (Bio-Rad, Hercules, CA, USA). Relative gene expression was calculated with the ΔΔCt method in the CFX96 Manager software (Bio-Rad, Hercules, CA, USA). The housekeeping gene *Gusβ* was used as reference control.

### 2.7. Protein Extraction and Western Blot

Protein extraction and Western blotting were performed as described [38]. Briefly, protein was extracted from snap-frozen liver tissue samples in lysis buffer (40 mM KCl, 25 mM Tris and 1% Triton X100, pH 7.4). After separation by standard sodium dodecylsulfate polyacrylamide gel electrophoresis (SDS-PAGE), the proteins were blotted onto a polyvinylidene fluoride (PVDF) membrane. After blocking the following antibodies were used: mouse TfR1 antibody (1:1000; Invitrogen, Waltham, MA, USA), rabbit ferritin antibody (1:500; Sigma-Aldrich, St. Louis, MO, USA), rabbit mitoferrin 2 (Mfrn2) antibody (1:1000; Bioss, Woburn, MA, USA), rabbit mitochondrial ferritin (MtF) antibody (1:1000; Abcam, Cambridge, United Kingdom), rabbit Fpn antibody (1:400; Eurogentec, Seraing, Belgium), rabbit β-Actin antibody (1:1000; Sigma-Aldrich, St. Louis, MO, USA), rabbit iron regulatory protein 2 (Irp2) antibody (1:1000; Novus Biological, Littleton, CO, USA), total rodent OXPHOS antibody cocktail (1:1000; Abcam, Cambridge, United Kingdom), mouse cytochrome c oxidase subunit 4 (CoxIV) antibody (1:1000, Abcam, Cambridge, United Kingdom), rabbit α-Tubulin antibody (1:1000, Cell Signaling Technology, Danvers, MA, USA) and appropriate horseradish peroxidase (HRP) conjugated secondary antibodies (1:4000; Dako North America, Carpinteria, CA, USA). The chemiluminescent reaction was performed using Pierce^TM^ SuperSignal West Dura Reagent (Thermo Fisher, Waltham, MA, USA). The blots were visualized by ChemiDoc device using Image Lab software (Bio-Rad, Hercules, CA, USA) and analyzed by ImageJ software (1.53k, NIH, Bethesda, MD, USA).

### 2.8. Aconitase and Citrate Synthase Activity

For aconitase activity determination, a commercially available assay was used (MAK051, Sigma-Aldrich, St. Louis, MO, USA). Samples were prepared according to the manufacturer’s instructions. Briefly, snap-frozen liver samples were homogenized in assay buffer and differentially centrifuged to obtain the mitochondrial and cytosolic subcellular fractions. The mitochondrial fraction was sonicated and both fractions were activated using the aconitase activation solution, a 1:1 mixture of cysteine and NH_4_Fe(SO_4_)_2_ on ice for 1 h. The activated samples were incubated with the reaction mix containing assay buffer, enzyme mix and substrate at 25 °C for 30 to 60 min. After addition of the developer and another 10 min incubation time at 25 °C, absorbance was measured at 450 nm. The resulting enzyme activities were normalized by the protein content of the samples.

Citrate synthase (CS) activity was measured as described [14]. A spectrophotometric assay was used to measure the enzyme activity in snap-frozen liver homogenates. The sample enzymatic reaction mix contained 0.25% Triton X-100 in aqua dest (a.d.), 0.31 mM acetyl-coenzyme A (acetyl-CoA) in a.d., 0.1 mM 5,50-dithiobis-(2-nitrobenzoic acid) (DTNB) in 1 M Tris–HCl buffer (pH 8.1) and 0.5 mM oxaloacetate in 0.1M triethanolamine–HCl-buffer (pH 8.0). The absorbance of the reaction product thionitrobenzoic acid (TNB) was measured at 412 nm over 200 s. The resulting enzyme activities were normalized by the protein content of the samples.

### 2.9. Lactate and ATP Concentration

Lactate concentration in the liver samples was measured using a commercially available assay (K607, BioVision, Milpitas, CA, USA) according to manufacturer’s protocol. Briefly, snap-frozen liver samples were homogenized in assay buffer and filtered through a 10 kDa molecular weight spin filter. The samples were incubated with a reaction mix consisting of enzyme mix and probe for 30 min at room temperature. Absorbance was measured at 570 nm. The resulting lactate concentrations were normalized by the protein content of the samples.

ATP concentration in the liver samples was determined using a commercially available assay (MAK190, Sigma-Aldrich, St. Louis, MO, USA) according to manufacturer’s protocol. Briefly, snap-frozen liver samples were homogenized in assay buffer and filtered through a 10 kDa molecular weight spin filter. The samples were incubated with a reaction mix consisting of probe, converter and developer mix for 30 min at room temperature. Absorbance was measured at 570 nm. The resulting ATP concentrations were normalized by the protein content of the samples.

### 2.10. Mitochondrial DNA Copy Number

Mitochondrial DNA copy number (mtDNA-CN) was determined following a protocol adapted from Fazzini et al. and Singh et al. [39,40]. Genomic DNA was extracted from snap-frozen liver pieces in 5% Chelex 100 resin (Sigma-Aldrich, St. Louis, MO, USA at 100 °C for 15 min under constant shaking at 500 rpm. After centrifugation at 12,000× *g* for 1.5 min the yield and purity of the DNA in the supernatant was quantified and a quantitative real-time PCR was performed using the following primer probe combinations: mitochondrial gene, *mtRnr2* forward CCTGCCCAGTGACTAAAGTT, mtRnr2 reverse GACAGTTGGACCCTCGTTTAG, *mtRnr2* probe ATCCTGACCGTGCAAAGGTAGCAT; nuclear gene, *Gusβ* forward GAGCTTTCGAAGCAGGAGTAG, *Gusβ* reverse CCAGGAGAGGTGAAGTGTTATG, *Gusβ* probe AGATGGACCACACTTCACAGGTCA. Real-time PCR reactions were performed on the CFX96 PCR System (Bio-Rad, Hercules, CA, USA). PCR efficiency was determined by 3-fold serial dilutions of the DNA; mtDNA copy number was calculated using the formula mtDNA-CN = 2 × E^ΔCt^. E = 1.9915, which is the qPCR efficiency calculated as the mean amplification factor of the two primer probe combinations and ΔCt equals Ct_nuclear_ gene—Ct_mitochondrial_ gene.

### 2.11. Flow Cytometry and Fluorescence Microscopy

Primary murine hepatocytes were isolated as described [41]. For flow cytometry, the cells were seeded in a density of 400,000 cells in 6-well plates and treated with 100 µM ferric maltol (Shield Therapeutics PLC) for 48 h. After washing, the cells were stained with 2.5 µM MitoSOX Red (Thermo Fisher, Waltham, MA, USA) for 15 min at 37 °C. Afterwards, the cells were washed, detached with Tryp-LE (Gibco, Darmstadt, Germany) and, after a further washing step, resuspended in FACS buffer (PBS supplemented with 0.5% fetal bovine serum and 2 mM EDTA) containing DAPI (1:50,000, Sigma-Aldrich, St. Louis, MO, USA). Data were acquired using the flow cytometer CytoFLEX s (Beckman Coulter, Brea, CA, USA) and analyzed with the FlowJo software (10.6.1, FlowJo LLC, Ashland, OR, USA).

For fluorescence microscopy, primary murine hepatocytes were seeded on cover slips (18 × 18 mm, VWR, Radnor, PA, USA) in a density of 400,000 cells in 6-well plates and treated as for flow cytometry. After staining with 2.5 µM MitoSOX Red (Thermo Fisher, Waltham, MA, USA) for 15 min at 37 °C, the cells were washed and fixed with 4% formaldehyde solution for 20 min at room temperature. After 3 washes, the cells were mounted in fluorescence mounting media (Agilent, Santa Clara, CA, USA) containing DAPI (1:10,000, Sigma-Aldrich, St. Louis, MO, USA). Images were acquired using a fluorescence microscope (IXplore Live, Olympus, Shinjuku, Japan) and analyzed with the ScanR software (Olympus, Shinjuku, Japan).

### 2.12. Statistics

Statistical analysis was carried out using the software GraphPad Prism 9 (GraphPad Software, San Diego, CA, USA). In case of normal distribution, statistical significance was determined by a two-way ANOVA with Tukey’s post-hoc test. Otherwise, a Kruskal–Wallis test with Dunn’s post-hoc test was applied. Data was shown as median ± interquartile range. *p*-values below 0.05 were considered significant (* *p* < 0.05; ** *p* < 0.01; *** *p* < 0.001).

## 3. Results and Discussion

### 3.1. Alteration of Systemic and Cellular Iron Metabolism by Dietary and Genetic Iron Overload

**Figure 1:**
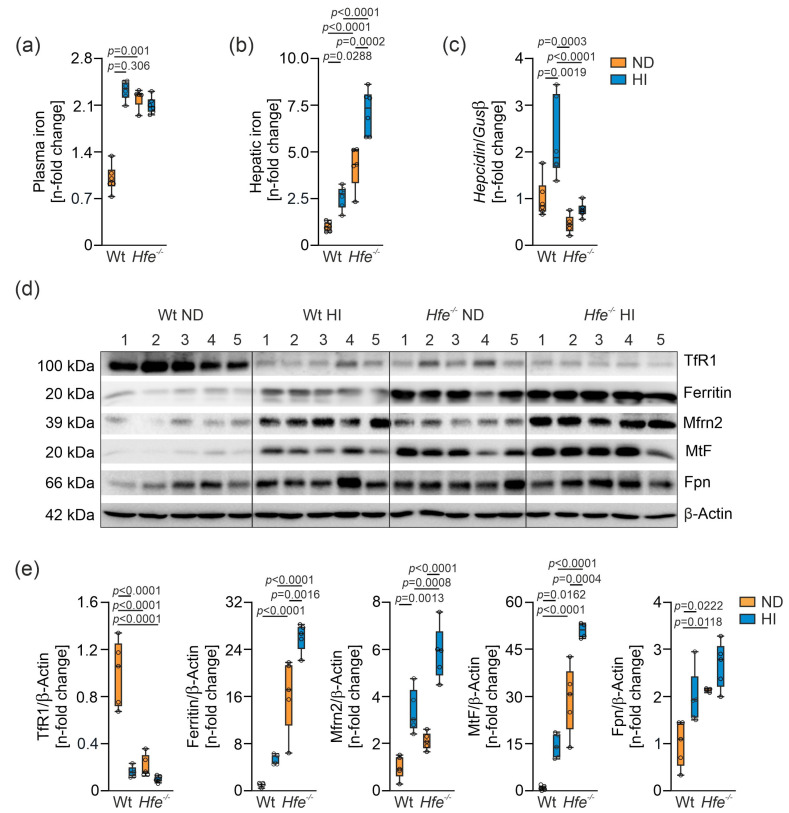
Impact of dietary and genetic iron overload in wildtype (Wt) and *Hfe^−/−^* mice on systemic and hepatic iron homeostasis. Wt and *Hfe^−/−^* mice were fed normal diet (ND) or high iron diet (HI) for two weeks. (**a**) Plasma iron concentration; (**b**) hepatic iron content; (**c**) hepatic hepcidin mRNA expression; (**d**) Western blots of proteins involved in cellular and mitochondrial iron metabolism (transferrin receptor 1 (TfR1), ferritin, mitoferrin 2 (Mfrn2), mitochondrial ferritin (MtF), ferroportin (Fpn)) along with (**e**) densitometric quantification of Western blots. β-Actin served as loading control. All results were obtained from snap-frozen samples. *n* = 5–6 mice per group. Values are depicted as n-fold change of Wt ND. Kruskal–Wallis test: (**a**) *p* = 0.0014; two-way ANOVA: (**b**) diet, F_(1,19)_ = 36.00, *p* < 0.0001; genotype, F_(1,19)_ = 108.8, *p* < 0.0001; diet X genotype, F_(1,19)_ = 3.062, *p* = 0.0963; (**c**) diet, F_(1,19)_ = 13.74, *p* = 0.0015; genotype, F_(1,19)_ = 23.60, *p* = 0.0001; diet X genotype, F_(1,19)_ = 5.067, *p* = 0.0364; (**e**) TfR1—diet, F_(1,16)_ = 48.86, *p* < 0.0001; genotype, F_(1,16)_ = 39.44, *p* < 0.0001; diet X genotype, F_(1,16)_ = 28.76, *p* < 0.0001; Ferritin—diet, F_(1,16)_ = 21.44, *p* = 0.0003; genotype, F_(1,16)_ = 147.3, *p* < 0.0001; diet X genotype, F_(1,16)_ = 3.433, *p* = 0.0825; Mfrn2—diet, F_(1,16)_ = 72.74, *p* < 0.0001; genotype, F_(1,16)_ = 25.05, *p* = 0.0001; diet X genotype, F_(1,16)_ = 3.673, *p* = 0.0733; MtF—diet, F_(1,15)_ = 39.71, *p* < 0.0001; genotype, F_(1,15)_ = 137.2, *p* < 0.0001; diet X genotype, F_(1,15)_ = 2.387, *p* = 0.1432; Fpn—diet, F_(1,15)_ = 12.19, *p* = 0.0033; genotype, F_(1,15)_ = 17.98, *p* = 0.0007; diet X genotype, F_(1,15)_ = 1.091, *p* = 0.3129. Values are shown as median ± interquartile range. The exact *p*-values are indicated in the graphs.

Wt and *Hfe^−/−^* mice received ND or HI for two weeks ad libitum to study the metabolic effects of dietary and/or genetic iron overload. As expected, plasma and liver tissue iron concentrations were increased in all groups compared to Wt mice on ND (Figure 1a,b). Moreover, hepcidin gene expression was elevated in Wt mice on ND compared to *Hfe^−/−^* mice on ND. HI diet increased *hepcidin* gene expression in Wt but not in *Hfe^−/−^* mice (Figure 1c). We then analyzed protein expression involved in cellular and mitochondrial iron metabolism by Western blot (Figure 1d,e). Hepatic TfR1 expression was decreased in all groups compared to Wt mice on ND, whereas cellular protein levels of the iron storage protein ferritin were increased in parallel to excess cellular iron availability. Furthermore, expression of the mitochondrial iron importer Mfrn2 was significantly increased in dietary iron overload in both genotypes and higher in *Hfe^−/−^* than in Wt mice on ND. MtF protein expression was elevated in direct proportion to ferritin. Protein expression of the cellular iron exporter Fpn was increased in dietary and in genetic iron overload.

### 3.2. Iron Content in Liver Mitochondria of Hfe^−/−^ mice was Increased and m-Aconitase Activity Was Decreased

**Figure 2:**
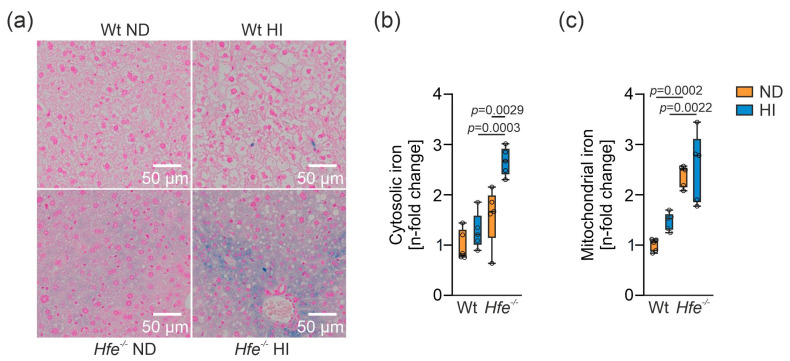
Cellular and mitochondrial iron content in livers of different treatment groups. (**a**) Histological iron staining in livers of Wt and *Hfe^−/−^* mice on ND and HI. Scale bar = 50 µm. (**b**) Cytosolic and (**c**) mitochondrial iron content in *Hfe^−/−^* mice on ND and HI. Cytosolic and mitochondrial iron concentrations were obtained from snap-frozen samples. *n* = 5 mice per group. Values are depicted as n-fold change of Wt ND. Two-way ANOVA: (**b**) diet, F_(1,16)_ = 14.53, *p* = 0.0015; genotype, F_(1,16)_ = 30.74, *p* < 0.0001; diet X genotype, F_(1,16)_ = 5.028, *p* = 0.0395; (**c**) diet, F_(1,16)_ = 3.535, *p* = 0.0784; genotype, F_(1,16)_ = 51.04, *p* < 0.0001; diet X genotype, F_(1,16)_ = 0.8050, *p* = 0.3829. Values are shown as median ± interquartile range. The exact *p*-values are indicated in the graphs.

To estimate liver iron distribution in the different treatment groups, Prussian blue staining was performed (Figure 2a). *Hfe^−/−^* mice showed iron accumulation in the tissue due to genetic iron loading, especially those receiving an additional HI [18,42]. To determine iron overload distribution in the liver cells, iron content was determined in the cytosol and in isolated crude mitochondria (Supplemental Figure S2). Cytosolic iron content was significantly elevated in *Hfe^−/−^* mice on HI compared to all other groups (Figure 2b). Mitochondrial iron content increased in *Hfe^−/−^* ND and HI, but iron content in *Hfe^−/−^* HI did not differ from *Hfe^−/−^* ND (Figure 2c), suggesting that *Hfe* deficiency already results in mitochondrial iron loading over time.

**Figure 3:**
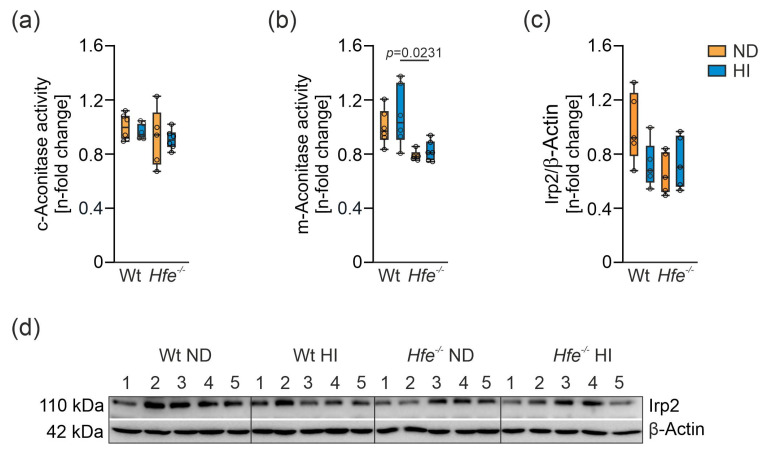
Functional effect of dietary and genetic iron overload on iron metabolism. (**a**) Cytosolic aconitase (c-aconitase) and (**b**) mitochondrial aconitase (m-aconitase) activity in *Hfe^−/−^* mice on ND and HI. (**c**) and (**d**), densitometric quantification and Western blot of iron regulatory protein 2 (Irp2) protein. β-Actin served as loading control. Values are depicted as n-fold change of Wt ND. All results were obtained from snap-frozen samples. *n* = 5–6 mice per group. Two-way ANOVA: (**a**) diet, F_(1,19)_ = 0.2176, *p* = 0.6462; genotype, F_(1,19)_ = 1.813, *p* = 0.1940; diet X genotype, F_(1,19)_ = 0.05023, *p* = 0.8251; (**b**) diet, F_(1,19)_ = 0.9945, *p* = 0.3312; genotype, F_(1,19)_ = 15.89, *p* = 0.0008; diet X genotype, F_(1,19)_ = 0.1675, *p* = 0.6869; (**c**) diet, F_(1,16)_ = 1.291, *p* = 0.2726; genotype, F_(1,16)_ = 3.135, *p* = 0.0957; diet X genotype, F_(1,16)_ = 4.116, *p* = 0.0595. Values are shown as median ± interquartile range. The exact *p*-values are indicated in the graphs.

To study the functional effect of iron overload on iron controlled metabolic pathways, aconitase activity was analyzed. Aconitase activity is regulated by cellular iron availability and exists in a cytosolic form as an iron sensing enzyme, also known as iron regulatory protein 1 (Irp1), and in a mitochondrial form as part of the TCA cycle which converts citrate to isocitrate [2,13]. Surprisingly, hepatic c-aconitase activity was not different between the various treatment groups (Figure 3a). We measured Irp2 expression by Western blot because Irp2 is degraded by metabolically active iron [43,44]. Of note, Irp2 levels were not different between the four treatment groups (Figure 3c), which agrees with unaltered c-aconitase activities (Figure 3a). This suggested that metabolically active iron levels in the cytoplasm were similar between the different groups and that surplus iron was sequestered within ferritin (Figure 1 and Figure 2). m-Aconitase activity decreased in *Hfe^−/−^* mice on ND and HI (Figure 3b), suggesting decreased function of the TCA cycle [12,14]. Decreased m-aconitase activity might be caused by increased ROS formation as a consequence of mitochondrial iron loading (Figure 2), resulting in its inactivation or decreased ability of antioxidant enzymes to scavenge ROS, possibly due to diminished MnSOD activity as shown in dietary and genetic iron overload [14,26,45,46].

### 3.3. Mitochondrial Respiration in Wt and Hfe^−/−^ Mice on ND and HI

**Figure 4:**
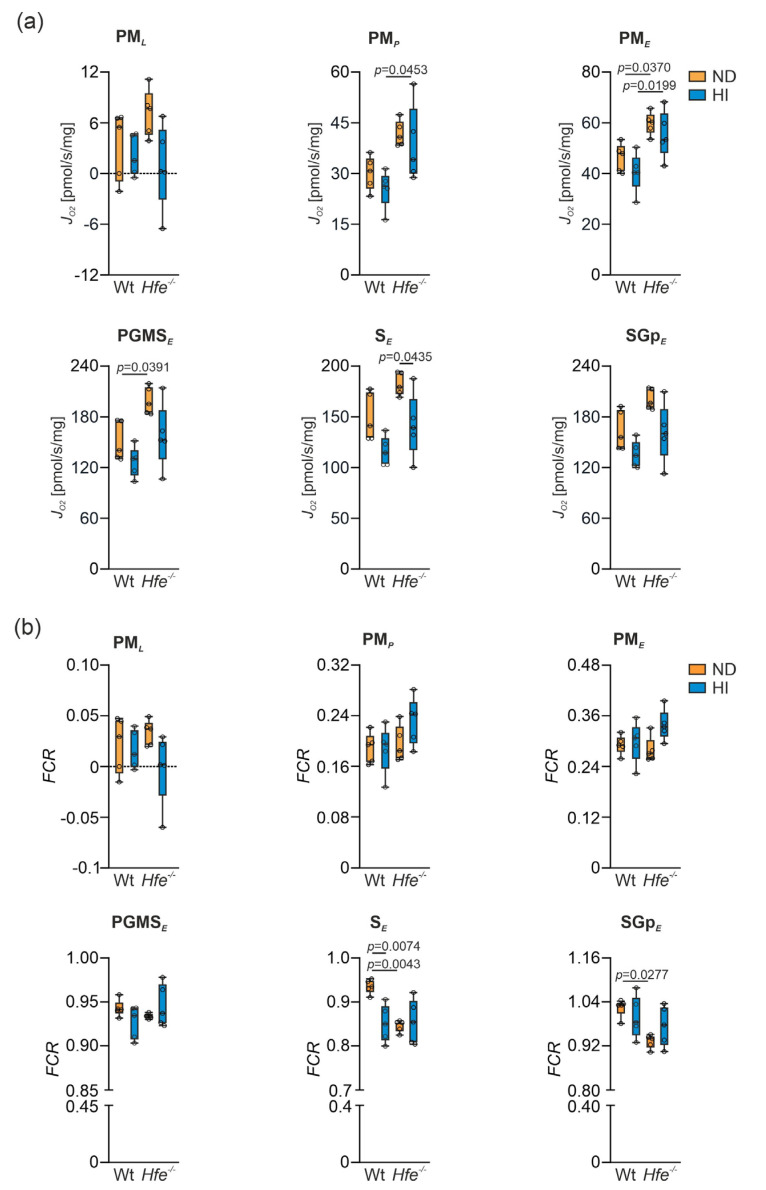
Effect of dietary and genetic iron overload on mitochondrial respiration in mouse liver. (**a**) Mitochondrial respiratory capacity in states PM*_L_*, PM*_P_*, PM*_E_*, PGMS*_E_*, S*_E_* and SGp*_E_* measured in homogenates from freshly isolated liver. PM*_L_*, non-phosphorylating LEAK respiration after addition of pyruvate and malate as NADH-(N) linked substrates; PM*_P_*, OXPHOS capacity upon addition of adenosine diphosphate (ADP) at kinetically saturating concentration; PM*_E_*, titrations of the protonophore carbonyl cyanide m-chloro phenyl hydrazine to reach the maximal respiration as a measure of electron transfer (ET) capacity; PGMS*_E_*, addition of succinate (S) provides the simultaneous action of N-linked substrates and S with convergent electron flow in the NS-pathway for reconstitution of tricarboxylic acid cycle function; S*_E_*, Complex I inhibition by rotenone for measurement of S-linked ET capacity; SGp*_E_*, titration of glycerophosphate (Gp) provided simultaneous action of S-linked substrate succinate and Gp with convergent electron flow in the SGp-pathway. (**b**) Flux control ratios (FCR) for PM*_L_*, PM*_P_*, PM*_E_*, PGMS*_E_*, S*_E_* and SGp*_E_* shown in (**a**); OctPGMS*_E_* served as common reference state. *n* = 5 mice per group. Two-way ANOVA: (**a**) PM*_L_*—diet, F_(1,16)_ = 5.109, *p* = 0.0381; genotype, F_(1,16)_ = 0.6326, *p* = 0.4380; diet X genotype, F_(1,16)_ = 2.315, *p* = 0.1476; PM*_P_*—diet, F_(1,16)_ = 1.595, *p* = 0.2247; genotype, F_(1,16)_ = 15.26, *p* = 0.0013; diet X genotype, F_(1,16)_ = 0.04508, *p* = 0.8345; PM*_E_*—diet, F_(1,16)_ = 2.602, *p* = 0.1263; genotype, F_(1,16)_ = 20.14, *p* = 0.0004; diet X genotype, F_(1,16)_ = 0.04877, *p* = 0.8280; PGMS*_E_*—diet, F_(1,16)_ = 8.285, *p* = 0.0109; genotype, F_(1,16)_ = 12.05, *p* = 0.0032; diet X genotype, F_(1,16)_ = 0.5708, *p* = 0.4609; S*_E_*—diet, F_(1,16)_ = 14.54, *p* = 0.0015; genotype, F_(1,16)_ = 8.936, *p* = 0.0087; diet X genotype, F_(1,16)_ = 0.1125, *p* = 0.7417; SGp*_E_*—diet, F_(1,16)_ = 10.37, *p* = 0.0053; genotype, F_(1,16)_ = 9.029, *p* = 0.0084; diet X genotype, F_(1,16)_ = 0.2804, *p* = 0.6037; (**b**) PM*_L_*—diet, F_(1,16)_ = 3.015, *p* = 0.1017; genotype, F_(1,16)_ = 0.07634, *p* = 0.7859; diet X genotype, F_(1,16)_ = 1.792, *p* = 0.1994; PM*_P_*—diet, F_(1,16)_ = 1.389, *p* = 0.2558; genotype, F_(1,16)_ = 3.054, *p* = 0.0997; diet X genotype, F_(1,16)_ = 1.714, *p* = 0.2090; PM*_E_*—diet, F_(1,16)_ = 4.189, *p* = 0.0575; genotype, F_(1,16)_ = 0.7642, *p* = 0.3949; diet X genotype, F_(1,16)_ = 2.707, *p* = 0.1194; PGMS*_E_*—diet, F_(1,15)_ = 0.06648, *p* = 0.8000; genotype, F_(1,15)_ = 0.4126, *p* = 0.5303; diet X genotype, F_(1,15)_ = 3.400, *p* = 0.0850; S*_E_*—diet, F_(1,16)_ = 5.711, *p* = 0.0295; genotype, F_(1,16)_ = 7.653, *p* = 0.0138; diet X genotype, F_(1,16)_ = 9.049, *p* = 0.0083; Kruskal–Wallis test: SGp*_E_*, *p* = 0.0385. Values are shown as median ± interquartile range. The exact *p*-values are indicated in the graphs.

To investigate the effect of dietary and genetic iron overload on liver mitochondrial function, mitochondrial respiration was measured in homogenate from freshly isolated livers using high-resolution respirometry (Supplemental Figure S1) [14,47]. In presence of the N-linked substrates P and M, a slight increase in O_2_ consumption in *Hfe^−/−^* mice compared to Wt ND was detected (Figure 4a, PM*_L_*). Furthermore, in presence of kinetically saturating concentrations of ADP, mitochondrial respiratory capacity was slightly decreased in Wt mice on HI but increased in *Hfe^−/−^* mice on ND and HI (Figure 4a, PM*_P_*). The same was observed in the non-coupled state PM*_E_* measuring ET capacity (Figure 4a, PM*_E_*). S-linked respiration in ET was significantly increased in *Hfe^−/−^* ND mice compared to Wt mice but not in Wt HI or *Hfe^−/−^* HI mice (Figure 4a, PGMS*_E_*) [14,28]. The same result was observed when Complex I was inhibited using Rot (Figure 4a, S*_E_*) and upon addition of Gp that provides simultaneous action of S-linked substrate succinate and Gp with convergent electron flow in the SGp-pathway (Figure 4a, SGp*_E_*).

**Figure 5:**
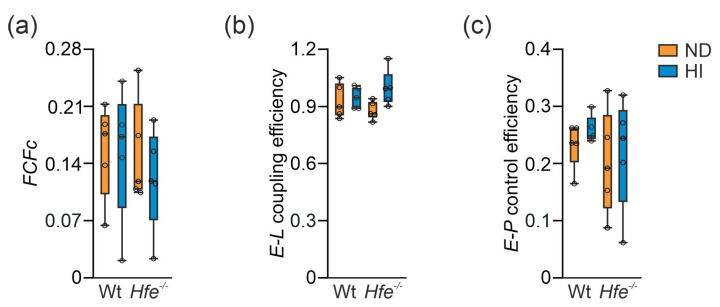
Impact of dietary and genetic iron overload on flux control factors. (**a**) Cytochrome c control factor (*FCFc* = 1-PM*_P_*/PM*_cP_*) indicates integrity of the mitochondrial outer membrane. (**b**) *E-L* coupling efficiency (*j_E-L_* = 1-PM*_L_*/PM*_E_*) indicates preserved coupling of electron transfer to phosphorylation of ADP. (**c**) *E-P* control efficiency (1-PM*_cP_*/PM*_E_*) indicates the limitation of OXPHOS capacity by the capacity of the phosphorylation system. *n* = 5 mice per group. Two-way ANOVA: (**a**) diet, F_(1,16)_ = 0.2989, *p* = 0.5921; genotype, F_(1,16)_ = 0.3760, *p* = 0.5484; diet X genotype, F_(1,16)_ = 0.2312, *p* = 0.6371; (**b**) diet, F_(1,16)_ = 3.875, *p* = 0.0666; genotype, F_(1,16)_ = 2.855 × 10^−6^, *p* = 0.9987; diet X genotype, F_(1,16)_ = 2.188, *p* = 0.1585; (**c**) diet, F_(1,16)_ = 0.5202, *p* = 0.4812; genotype, F_(1,16)_ = 1.240, *p* = 0.2819; diet X genotype, F_(1,16)_ = 0.01947, *p* = 0.8908. Values are shown as median ± interquartile range.

These results suggested mitochondrial adaptation to dietary and genetic iron overload. To investigate this in more detail, flux control ratios (*FCR*) were calculated by normalization of mitochondrial respiration for a common reference state (OctPGMS*_E_*), for expressing respiration independent of mitochondrial density (Figure 4b) [7,14]. As the *FCR* did not show major differences between the groups, mitochondrial damage can be excluded. Similarly, the cytochrome c control factor (*FCFc*, Figure 5a) was unaltered between the different groups. *FCFc* indicates the integrity of the mitochondrial outer membrane, which did not differ among the groups and therefore was not influenced by dietary or genetic iron overload [14]. Moreover, dietary and genetic iron overload did not affect *E-L* coupling efficiency and no differences in *E-P* control efficiency were detected, indicating oxidative phosphorylation was no limiting factor due to iron overload (Figure 5b,c) [14,48].

**Figure 6:**
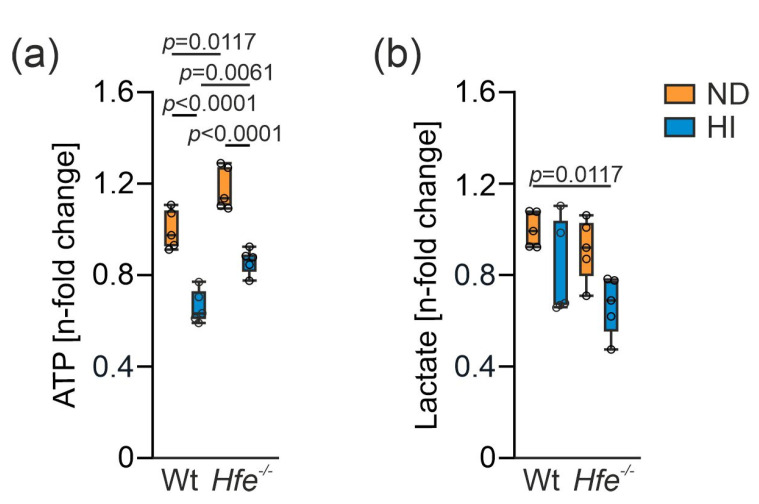
Changes in liver adenosine triphosphate (ATP) and lactate concentration due to dietary and genetic iron overload. (**a**) ATP and (**b**) lactate concentration. Values are depicted as n-fold change of Wt ND. All results were obtained from snap-frozen samples. *n* = 5 mice per group. Two-way ANOVA: (**a**) diet, F_(1,16)_ = 88.29, *p* < 0.0001; genotype, F_(1,16)_ = 28.17, *p* < 0.0001; diet X genotype, F_(1,16)_ = 0.05114, *p* = 0.8240; (**b**) diet, F_(1,16)_ = 10.71, *p* = 0.0048; genotype, F_(1,16)_ = 3.268, *p* = 0.0895; diet X genotype, F_(1,16)_ = 0.2411, *p* = 0.6301. Values are shown as median ± interquartile range. The exact *p*-values are indicated in the graphs.

Hepatic ATP concentrations indicated that excess dietary iron leads to decreased ATP levels, whereas genetic iron loading even moderately increased the amount of ATP (Figure 6a). As changes in mitochondrial function or aconitase activity may impact on metabolite composition [12,49,50], we determined lactate concentrations as an indicator of aerobic glycolysis. Iron loading in *Hfe^−/−^* mice resulted in lower lactate levels which indicate metabolic reprogramming of glucose homeostasis and the TCA cycle (Figure 6b) [12,31,51,52].

### 3.4. Liver Mitochondrial Density Was Unaltered but Protein Expression of Electron Transfer Complexes Was Slightly Upregulated in Genetic Combined with Dietary Iron Overload

**Figure 7:**
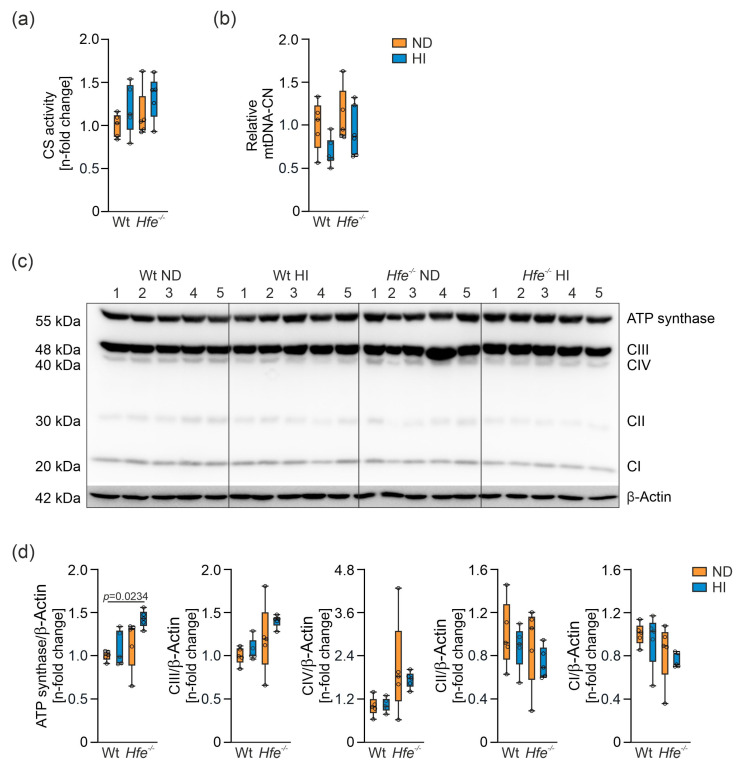
Effects of dietary and genetic iron overload on liver mitochondrial density and protein expression of mitochondrial electron transfer complexes I to IV (CI to CIV) and ATP synthase. (**a**) Citrate synthase (CS) activity, (**b**) relative mitochondrial DNA copy number (mtDNA-CN), (**c**) Western blot of C I to CIV and ATP synthase and (**d**) densitometric quantification of CI to CIV and ATP synthase relative to the house keeping protein β-Actin which served as control. Values are depicted as n-fold change of Wt ND. All results were obtained from snap-frozen samples. *n* = 5–6 mice per group. Kruskal–Wallis test: (**a**) *p* = 0.2933; two-way ANOVA: (**b**) diet, F_(1,18)_ = 4.479, *p* = 0.0485; genotype, F_(1,18)_ = 2.303, *p* = 0.1465; diet X genotype, F_(1,18)_ = 0.4127, *p* = 0.5287; (**d**) Kruskal–Wallis test: ATP synthase— *p* = 0.0183; two-way ANOVA: CIII—diet, F_(1,16)_ = 1.788, *p* = 0.1998; genotype, F_(1,16)_ = 7.107, *p* = 0.0169; diet X genotype, F_(1,16)_ = 0.4672, *p* = 0.5040; CIV—diet, F_(1,16)_ = 0.2165, *p* = 0.6480; genotype, F_(1,16)_ = 7.756, *p* = 0.0132; diet X genotype, F_(1,16)_ = 0.3278, *p* = 0.5749; CII—diet, F_(1,16)_ = 1.472, *p* = 0.2427; genotype, F_(1,16)_ = 0.9855, *p* = 0.3356; diet X genotype, F_(1,16)_ = 0.04946, *p* = 0.8268; CI—diet, F_(1,16)_ = 0.6000, *p* = 0.4499; genotype, F_(1,16)_ = 4.016, *p* = 0.0623; diet X genotype, F_(1,16)_ = 0.01808, *p* = 0.8947. Values are shown as median ± interquartile range. The exact *p*-values are indicated in the graphs.

Due to the observed changes in mitochondrial respiration without indication of damage of mitochondria, mitochondrial density was investigated. CS catalyzes the conversion of oxaloacetate and acetyl-CoA to citrate in the mitochondrial matrix. Its enzyme activity serves as a marker for the cellular content of mitochondria [53,54,55], but its general significance must be critically evaluated [8,9]. Dietary and genetic iron overload did not result in any significant changes in CS activity (Figure 7a). Moreover, relative mtDNA-CN, another surrogate marker for mitochondrial density, was statistically unaltered (Figure 7b). These results indicate that changes in mitochondrial respiratory capacity induced by dietary and genetic iron overload did not result from alterations in mitochondrial density.

Therefore, changes inside the organelle were investigated by analysis of protein expression of the electron transfer complexes I to IV and the ATP synthase to prove whether iron overload has a direct impact on the protein expression of the complexes [56]. Only ATP synthase in *Hfe^−/−^* mice on HI revealed an upregulation in its protein expression (Figure 7c,d). This seems to be a mechanism to compensate for the decreased mitochondrial respiratory capacity by increasing the amount of this complex [57]. Furthermore, upregulation of the protein expression could be a compensatory mechanism for the decreased m-aconitase activity that suggests a decreased availability of the reducing equivalent NADH for the ETS.

### 3.5. Dietary and Genetic Iron Overload Increased Liver Mitochondrial Superoxide Formation

**Figure 8:**
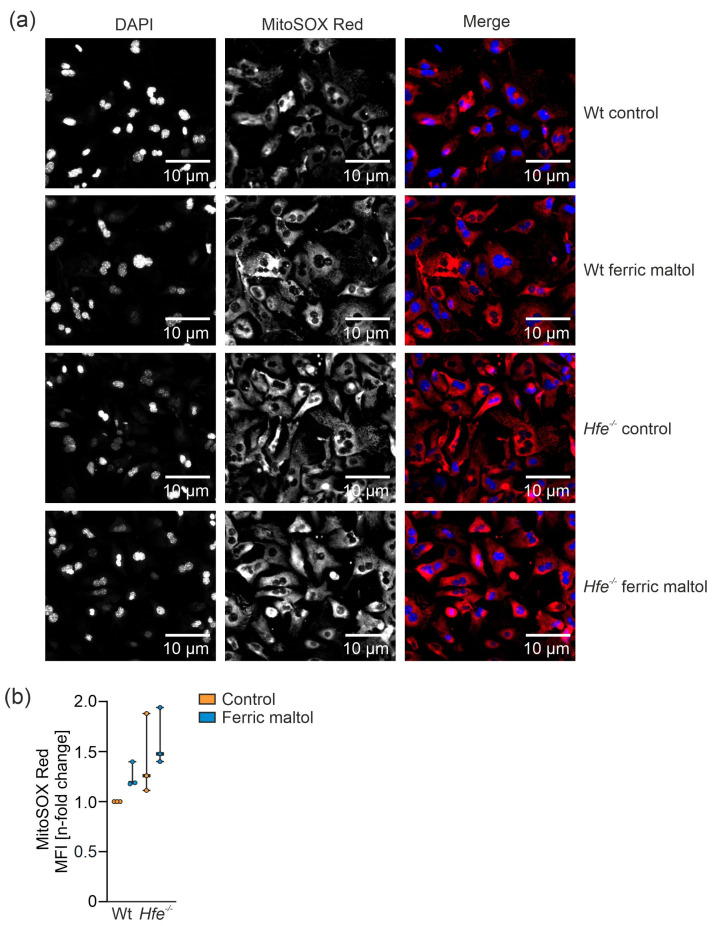
Determination of mitochondrial superoxide formation in primary murine hepatocytes from Wt and *Hfe^−/−^* mice in the presence or absence of the iron source ferric maltol (100 µM for 48 h). (**a**) Immunofluorescence images of primary murine hepatocytes isolated from Wt and *Hfe^−/−^* mice treated with 100 µM ferric maltol for 48 h were stained with MitoSOX (red; mitochondrial superoxide) and DAPI (blue; nuclei). Scale bar = 10 µm. (**b**) Flow cytometry analysis of mitochondrial superoxide formation in primary murine hepatocytes; the graph summarizes three independent experiments performed in triplicates (*n* = 3 mice per group, *n* = 3 replicates). MFI: mean fluorescence intensity. Two-way ANOVA: (**b**) Ferric maltol, F_(1,8)_ = 2.227, *p* = 0.1740; genotype, F_(1,8)_ = 6.608, *p* = 0.0331; ferric maltol X genotype, F_(1,8)_ = 0.04869, *p* = 0.8309. Values are shown as median ± interquartile range.

To investigate the link between changes in mitochondrial respiration and oxidative stress, we studied mitochondrial superoxide formation in primary murine hepatocytes from Wt and *Hfe^−/−^* mice in the presence or absence of ferric maltol serving as an iron source (Figure 8a,b). In dietary and genetic iron overload, mitochondrial superoxide production was elevated compared to Wt mice on ND. This might be explained by an increased iron concentration in mitochondria along with enhanced radical formation via the catalytic function of this metal [58] or decreased activity of radical detoxifying enzymes such as MnSOD [14,26]. Of note, *Hfe^−/−^* mice on ND showed increased mitochondrial superoxide production, even without additional dietary iron loading. This might have been caused by the elevation in mitochondrial respiratory capacity as Complexes I and III are able to produce increased radical amounts [59].

### 3.6. Summary

In summary, our investigation uncovered multiple and partly divergent effects of dietary and genetic iron loading on cellular and mitochondrial iron accumulation and mitochondrial function. While some of these effects may be attributed to iron accumulation, it appears that *Hfe*-mediated iron loading exerts specific effects on the activity of the electron transfer complexes. Moreover, cells appear to be well suited to cover increased iron accumulation by largely detoxifying metabolically reactive iron via incorporation into ferritin and MtF [60]. HH, however, leads to mitochondrial iron accumulation as reflected by increased MtF expression, likewise a measure to scope with increased radical formation due to increased mitochondrial respiratory capacity. In dietary iron overload, we found slightly decreased hepatic mitochondrial respiration and decreased ATP production, whereas in genetic iron overload mitochondrial respiration and ATP production were increased. The most plausible explanation, according to the results in the present study and literature, is increased mitochondrial ROS formation combined with reduced ROS scavenging in acute dietary iron overload [14,26,27,28]. In addition, no alteration in mitochondrial density was observed, neither by analysis of mtDNA-CN nor by CS activity and protein expression of the electron transfer complexes. Only in *Hfe*- mice on HI did the ATP synthase show increased protein expression, likewise a mechanism to scope with the additional excess iron on top of the anyways high iron availability and the decreased mitochondrial respiratory capacity. Therewith, the decreased lactate concentration in combined dietary and genetic iron overload may be explained by lactate being used for hepatic glucose production via gluconeogenesis or pyruvate synthesis to fuel the TCA cycle [31,51,52]. Nonetheless, increased mitochondrial superoxide production, as observed in *Hfe^−/−^* mice, might be the cause for the chronic fatigue that hemochromatosis patients suffer from. This provides an explanation why the reduction in body iron content by phlebotomy in patients reduces fatigue [14,20,22], a notion which needs to be experimentally verified. Additionally, m-aconitase activity was decreased in *Hfe^−/−^* mice which may be a negative effect of the increased formation of mitochondrial ROS [61,62]. The mechanism for how iron trafficking between cytosol and mitochondria is regulated and how mitochondrial iron homeostasis is regulated are yet to be clarified [3,63].

Alongside current literature, the results shown in this study provide evidence that dietary and genetic iron overload cause major changes and adaptations in mitochondrial metabolism, whereby a clear distinction is required between iron overload being acquired or caused by the *Hfe* gene mutation [14,31,49,64].

## 4. Conclusions

In conclusion, dietary and genetic iron loading induced liver mitochondrial iron overload, affected mitochondrial metabolic pathways and increased formation of mitochondrial superoxide. This might be the cause for the fatigue symptoms experienced by patients with genetic or secondary iron overload. In contrast to mice showing acute dietary iron overload, *Hfe^−/−^* mice on ND seem to be genetically adapted to chronic exposure to excess iron and, consequently, did not show impairment in mitochondrial respiratory capacity but a need for increased mitochondrial respiration. Reducing iron levels in genetic iron loading may positively affect mitochondrial function and subsequent metabolic traits.

## Data Availability

All data presented within this study are available within the manuscript and the Supplementary Materials.

## Supplementary Materials

The following are available online at https://www.mdpi.com/article/10.3390/antiox10111818/s1, Figure S1: Representative trace of substrate-uncoupler-inhibitor titration (SUIT) protocol for measurement of liver mitochondrial respiration using high-resolution respirometry, Figure S2: Representative Western blot of isolated liver fractions to ensure their purity.
