# Supplementary material for: Dietary Iron Overload and Hfe−/− Related Hemochromatosis Alter Hepatic Mitochondrial Function
Source: Antioxidants (Basel). 2021 Nov 16;10(11):1818. doi: 10.3390/antiox10111818 (PMC8615072; doi:10.3390/antiox10111818)
Supplement: Supplementary file 1 [file antioxidants-10-01818-s001.zip › antioxidants-1424698-supplementary.pdf]

Supplementary Materials:

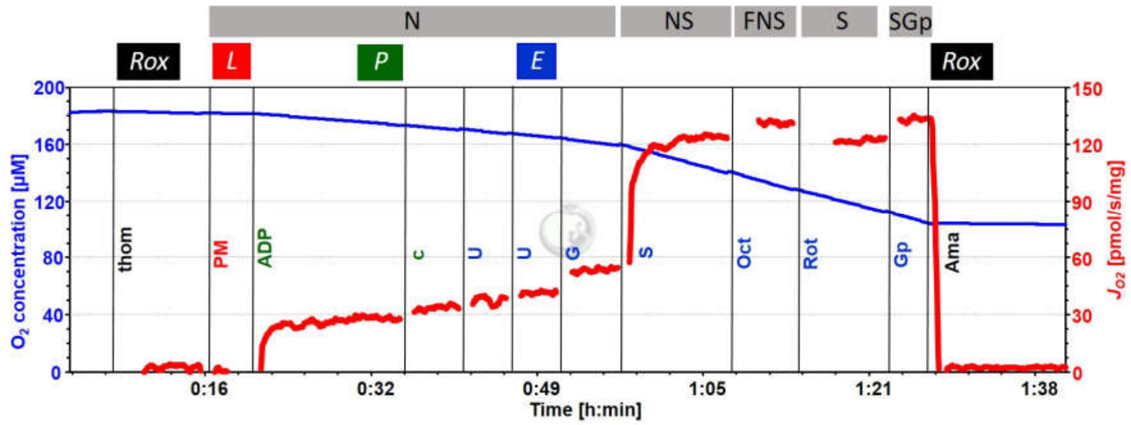

Figure S1. Representative trace of substrate-uncoupler-inhibitor titration (SUIT) protocol for measurement of liver mitochondrial respiration using high-resolution respirometry. Residual oxygen consumption (*Rox*) measured in the presence of liver homogenate (thom); pyruvate and malate (PM) in the absence of adenosine diphosphate (ADP) to measure NADH-linked (N) LEAK-respiration (*L*); kinetically saturating concentration of ADP to measure OXPHOS-capacity (*P*); cytochrome c (c) to detect mitochondrial outer membrane integrity; uncoupler titrations (U) to measure electron transfer (ET) capacity (*E*); glutamate (G) to measure N-linked ET capacity; succinate (S) to measure NS-linked ET capacity; octanoylcarnitine (Oct) to detect FNS-linked ET capacity; Complex I inhibitor rotenone (Rot) to measure S-ET capacity; glycerol-3-phosphate (Gp) to measure SGp-ET capacity; antimycin A (Ama) to detect *Rox*. Experiment 2021-02-01 PS1-01 WT ND 1: O_2 concentration (blue trace) and O_2 flux per mass (red trace).

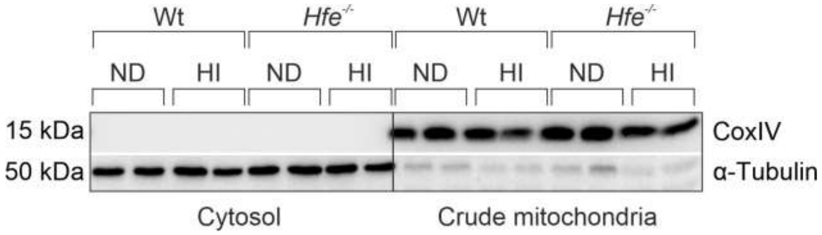

Figure S2. Representative Western blot of isolated liver fractions to ensure their purity. Mitochondrial protein cytochrome c oxidase subunit 4 (CoxIV) and cytosolic protein α-Tubulin.
